# Modeling an ultra-rare epilepsy variant in wildtype mice with in utero prime editing

**DOI:** 10.1101/2023.12.06.570164

**Published:** 2023-12-08

**Authors:** Colin D. Robertson, Patrick Davis, Ryan R. Richardson, Philip H. Iffland, Daiana C. O. Vieira, Marilyn Steyert, Paige N. McKeon, Andrea J. Romanowski, Garrett Crutcher, Eldin Jašarević, Steffen B. E. Wolff, Brian N. Mathur, Peter B. Crino, Tracy L. Bale, Ivy E. Dick, Alexandros Poulopoulos

**Affiliations:** 1 Department of Pharmacology and UM-MIND, University of Maryland School of Medicine, Baltimore, MD, USA; 2 Department of Neurology, Boston Children’s Hospital, Harvard Medical School, Boston, MA, USA; 3 Department of Neurology, and UM-MIND, University of Maryland School of Medicine, Baltimore, MD, USA; 4 Department of Physiology and UM-MIND, University of Maryland School of Medicine, Baltimore, MD, USA

## Abstract

Generating animal models for individual patients within clinically relevant time frames holds the potential to revolutionize personalized medicine for rare genetic epilepsies. By incorporating patient-specific genomic variants into model animals, capable of replicating elements of the patient’s clinical manifestations, a range of applications would be enabled: from preclinical platforms for rare disease drug screening, to bedside surrogates for tailoring pharmacotherapy without subjecting the patient to excessive trial medications. Here, we present the conceptual framework and proof-of-principle modeling of an individual epilepsy patient with an ultra-rare variant of the NMDA receptor subunit GRIN2A. Using in utero prime editing in the embryonic brain of wildtype mice, our approach demonstrated high editing precision and induced frequent, spontaneous seizures in prime editor-treated mice, reflecting key features of the patient’s clinical presentation. Leveraging the speed and versatility of this approach, we introduce PegAssist, a generalizable 7-week workflow for bedside-to-bench modeling of patients using in utero prime editing. These individualized animal models can allow for widely-accessible personalized medicine for rare neurological conditions, as well as accelerate the drug development pipeline by providing an efficient and versatile preclinical platform for screening compounds against ultra-rare genetic diseases.

## Introduction

Genetic epilepsies exhibit significant etiological heterogeneity, with nearly 1000 associated gene variants identified and corresponding diversity in clinical manifestations (1, 2). While prevalent in aggregate, genetic epilepsies largely comprise ultra-rare variants (3) for which treatment options are limited due to challenges in assembling large study trial cohorts.

More than half of all epilepsy patients require multiple trials of medications and approximately 30% of patients nonetheless remain resistant to pharmacotherapy (4). Even in patients with a known genetic cause of epilepsy, reliable prediction of therapeutic or deleterious response to medication trials remains elusive (2). In this space of inadequately treated ultra-rare epilepsies, a platform to identify patient-specific efficacies through existing anti-epileptics or the off-label use of compounds approved for human use would offer a path toward systematizing treatment selection in a manner that would otherwise not be clinically feasible.

For personalized medicine approaches to be applicable in a clinical setting, the technology for producing personalized model animals needs to be i) rapid, applicable in clinically relevant time scales, ii) versatile, applicable to a range of patients with distinct genetic variants, and iii) validatable, able to recapitulate identifiable features of the individual’s clinical presentation that are measurable against therapeutic interventions.

**Fig. 1.**
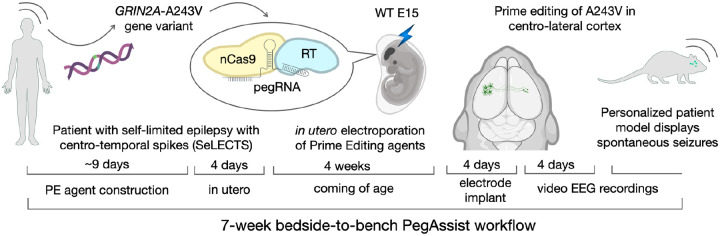
“PegAssist” personalized animal model workflow. Schema depicting the 7-week PegAssist workflow for personalized animal models, beginning from variant identification, and including genome editing agent construction, in utero delivery, and baseline analysis of personalized animal models.

We present PegAssist, an experimental approach which demonstrates these features by leveraging developments in somatic cell genome editing and new precision editors (5–7) in a workflow applicable within weeks (Fig. 1). We used prime editing in utero to genomically introduce an epilepsy patient point-variant into the brain of individual wildtype mice. The PegAssist platform produced individualized animal models that displayed frequent, spontaneous seizures reproducing several core characteristics of the clinical presentation of the patient.

## Prime editing 3b demonstrates high editing precision

**Fig. 2.**
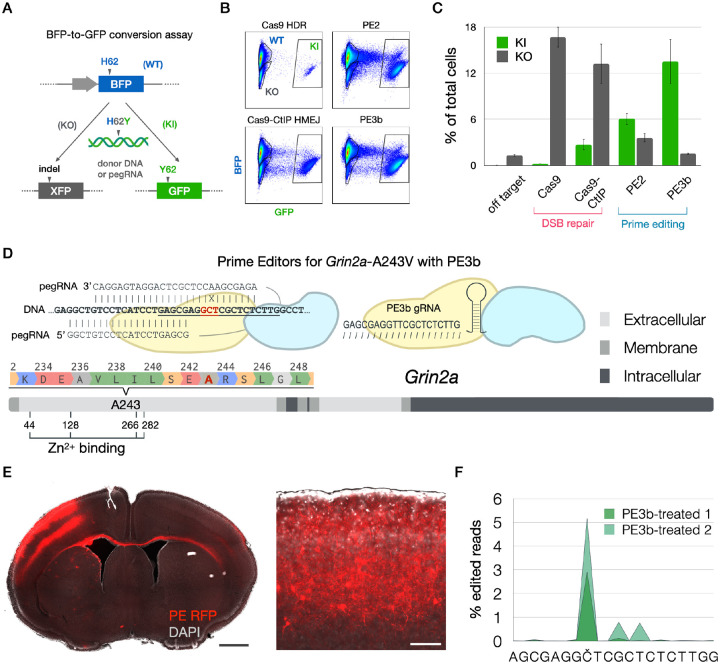
Genomic insertion of epilepsy patient variant of *GRIN2A* with in utero Prime editing. (**A**) Schema of BFP-to-GFP conversion assay to assess editor precision. (**B**) FACS plots and (**C**) quantification of blue unedited (WT), green correctly edited (knock-in; KI), and dark incorrectly edited (knock-out; KO) HEK cells after treatment with double-strand-break (DSB) repair editors Cas9 (with homology directed recombination [HDR] template) and Cas9-CtIP (with homology-mediated end-joining [HMEJ] template), and prime editing strategies PE2 and PE3b. PE3b outperforms other editing strategies through both higher KI rates and lower KO rates. (**D**) Alignment and schema of the WT *Grin2A* target sequence with the pegRNA and PE3b gRNA used to introduce edit A243V. Underlined is the sequence targeted by the PE3b gRNA. In red is the codon for A243. X shows the mismatch between target sequence and pegRNA RT template that introduces the A243V edit. The grey scale bar represents GRIN2A protein primary sequence with cellular topology as indicated in the key. Edited residue A243 and critical residues for Zn^2+^-binding are indicated. (**E**) Coronal section of DAPI-stained (grey) brain electroporated with PE and fluorescent marker (red) in centro-lateral cortex. Magnified inset shows electroporated upper-layer pyramidal neurons expressing PE. Scale bar = 1 mm, inset 100 μm. (**F**) Sequencing plot from fluorescence-sorted neurons from PE3b-treated mice showing the percentage of sequencing reads that deviated from the reference sequence around the genomic target sequence encoding A243. The intended nucleotide to be edited is marked as Č. Editing predominantly occurs on the intended base.

We began by screening available and engineered high-performance genome editors (8, 9) for high on-target precision, assessed by the rate of intended over unintended edits on the genome. Precision is the key limiting parameter for in vivo somatic cell genome editing, in order to introduce the intended edit in the body without accumulating unintended loss-of-function edits (6). We assessed on-target precision by point editing a genomically-encoded Blue Fluorescent Protein (BFP) gene with agents to introduce the substitution H62Y, which corresponds to the sequence for Green Fluorescent Protein (GFP). Precise editing would convert BFP to GFP, while on-target loss-of-function errors (e.g.: indels) would result in loss of fluorescence (Fig. 2A and fig. S1).

With this strategy we quantified editing precision of homology-directed recombination (HDR) and homology-mediated end-joining (HMEJ) strategies with Cas9 and Cas9-CtIP (8), as well as of reverse transcriptase-mediated editing with Prime Editor (PE) and PE fused to hRad51 in both PE2 and PE3b strategies (9) (fig. S1b–c). This screen demonstrated exceptionally high precision of point editing with the PE3b strategy, yielding the correct edit over 5-fold more frequently than the aggregate of all other edits (Fig. 2B–C).

PE is a hybrid ribonucleoprotein consisting of a protein fusion of Cas9 nickase (nCas9) and reverse transcriptase (RT), in complex with a hybrid pegRNA consisting of a “spacer” sequence for nCas9 targeting, a “primer binding sequence” that hybridizes with nicked genomic DNA, and template sequence for RT to encode the edit. To employ the PE3b strategy, an independent gRNA with no RT component directs PE to nick the complementary strand to encourage productive pegRNA-mediated editing (Fig. 2D). To facilitate the design and production of PE agents, we created pegassist.app, a python-based webtool and plasmid set offered through Addgene (fig. S2 and Methods). This webtool may be used in tandem with other pegRNA design tools to optimize editing agent production (11, 12).

## In utero prime editing of GRIN2A variant from epilepsy patient

The exceptional performance of PE3b prompted us to explore its use directly in vivo to model an individual patient variant in wildtype mice. We selected to model a patient with self-limited epilepsy with centrotemporal spikes (SeLECTS) reported with an ultra-rare missense variant, A243V, in the *GRIN2A* gene, encoding the 2A subunit of the N-methyl-D-aspartate (NMDA) type glutamate receptor (13). GRIN genes are hotspot loci with hundreds of ultra-rare loss- and gain-of-function variants identified to cause conditions collectively termed GRINopathies that commonly present with seizures that range in severity and manifestation (14). Importantly, *Grin2a* knockout mice do not have spontaneous seizures (15). The considerable genotypic and phenotypic diversity among patients with GRIN2-related disorders (16) further highlights the importance of modeling patient-specific variants in animal models (3, 17).

We constructed PE3b agents to edit the A243V patient variant into the *Grin2a* locus of the mouse using pegassist.app (Fig. 2D and fig. S2). PE and fluorescent reporter plasmids were injected into the lateral telencephalic ventricle of E15 mouse embryos and targeted by in utero electroporation to upper layer pyramidal neurons in centrolateral cortex (Fig. 2E), broadly analogous to the area of centrotemporal cortex, where epileptiform activity is detected in patients with SeLECTS. Animals electroporated in utero with either PE or control plasmids came to term and were allowed to reach adulthood in their home cage.

We directly assessed editing performance in vivo in two PE3b-treated mice by dissociating and sorting fluorescent cells from the electroporated target area of cortex. RNA sequenced from sorted cells showed moderate editing efficiency, but high editing precision: the A243V edit was present in ~5% of reads, while less than 1% of reads displayed any on-target errors (Fig. 2F and fig. S3).

This performance is similar to the >5-fold prevalence of precise edits we observed for PE3b editing in vitro (Fig. 2C), and corresponds to orders-of-magnitude higher precision than other knockin approaches we previously tested (10). Further, this likely is an underestimate of editing precision, since substitutions appear in sequencing reads as technical artifacts, e.g. due to RT or PCR amplification errors during library preparation (18), which we did not attempt to discriminate from true editing errors. Importantly, the rate of insertion / deletion events (indels) was minimal (<0.001%), indicating that loss-of-function effects are not a significant editing outcome.

We additionally confirmed using electrophysiology that PE-electroporated neurons do not display *Grin2a* loss of function. NMDA currents of PE-electroporated neurons in culture were largely normal, unlike Cas9-electroporated *Grin2a* knockout neurons, which displayed pronounced reduction in Zn2+ blockade of NMDA currents (fig. S4), as expected by *Grin2a* loss of function (19). Using exogenous expression in HEK cells, we corroborated that the *Grin2a*-A243V variant does not measurably alter gating effects of Zn2+, in contrast to a previous report in oocytes (13). Taken together, these data show that our in utero PE3b strategy successfully incorporated the patient variant into the *Grin2a* locus in vivo, without detectable loss of function.

## In utero prime edited “PegAssist” mice carrying patient variant display spontaneous seizures

Having confirmed in vivo editing in a subset of neurons in centrolateral cortex of wildtype mice, we proceeded to monitor 7 PE-electroporated “PegAssist” (PA) and 6 control-electroporated (CT) mice using video-EEG for 96 hours to determine whether animals present any pathological features associated with SeLECTS. 3 of 7 PA animals displayed spontaneous seizures with behavioral and electrographic features similar to those seen in SeLECTS patients (Fig. 2A, fig. S5, and movies S1–4) (20, 21).

**Fig. 3.**
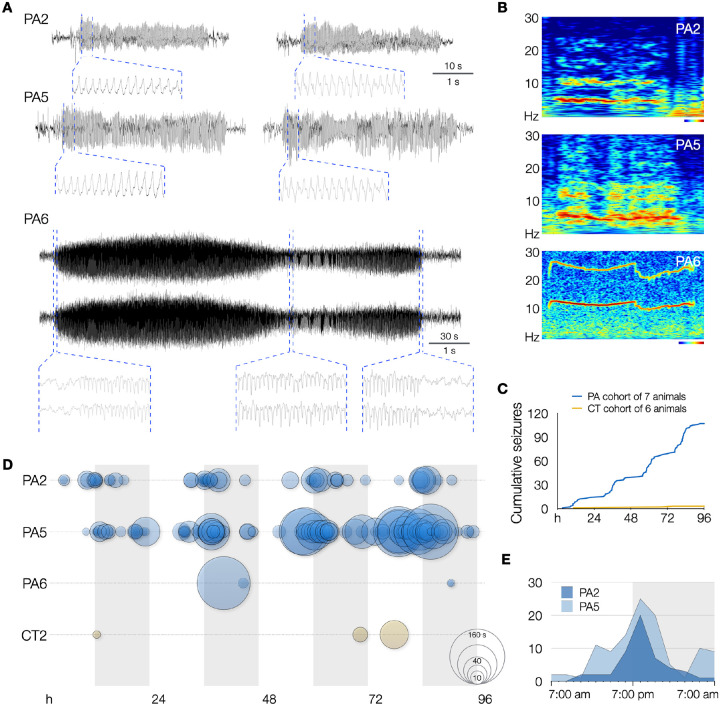
PegAssist *Grin2a*-A243V mice develop frequent spontaneous seizures. (**A**) Representative EEG traces from 3 of 7 PegAssist animals (PA2, PA5, and PA6) that developed spontaneous seizures after in utero prime editing with *Grin2a*-A243V. PA2 and PA5 developed frequent focal seizures, while PA6 presented a generalized seizure (EEG traces of both hemispheres shown) and sparse epileptiform events. Insets display magnifications of the indicated positions, showing spike-and-wave morphologies. Scale bars as indicated for full traces (top values) and insets (bottom values). (**B**) Morlet wavelet spectrograms of seizures in (a) showing characteristic dominant frequency bands and harmonics. Heatmap and scale bars = 10 s (PA2 and PA5) and 30 s (PA6). (**C**) Cumulative histogram of seizures over a 4-day recording period from PegAssist (PA; N=7) and control (CT; N=6) animals. Steps in cumulative histogram of PA cohort suggest circadian periodicity of seizures. (**D**) Seizures (solid circles) and epileptiform events (dashed circles) for each animal plotted by time and duration over 4-day recording period. Circle size indicates duration as indicated on the bottom right reference circles (10, 20, 40, 80, 160 s). Grey vertical bars indicate daily dark cycle. (**E**) Circadian histogram of seizures by hour in animal PA2 (dark blue) and PA5 (light blue). Seizures cluster in the 7:00–8:00 pm interval, corresponding to the beginning of the dark cycle when mice typically awaken.

Two of the PA animals (PA2 and PA5) showed frequent, spontaneous motor seizures associated with asymmetric tonic posturing with hemiclonic movements (movies S1–4). Focal motor and secondarily generalized seizures are both typical of patients with SeLECTs. As shown in the representative traces in Fig. 3A, events in PA2 and PA5 were electrographically characterized by sharply contoured, evolving spike-and-wave discharges in the ~5 Hz range. As evident in example spectrograms and averaged traces (Fig. 3B and fig. S5), these events have discrete onset and termination with consistent frequencies. This event type represented the majority of observed seizures. A second seizure type was observed in animal PA6, a single generalized electrographic seizure without a motor component occurring during sleep (Fig. 3A, PA6).

Aggregating events within groups after blinded review of video and EEG recordings over 4 days, the PA cohort had a total of 107 seizures and an additional 56 epileptiform events, compared to 3 total events classified as seizures from one animal (CT3) electroporated with Cas9 and scrambled gRNA from the control cohort (Fig. 3C, fig. S5, and table S1). We anticipated that only a subset of PA animals would manifest phenotypes due to the known variability of electroporation between individually treated embryos. For the PegAssist workflow, we propose that the treated animal cohort be segmented into spontaneously symptomatic and nonsymptomatic animals. Symptomatic animals would then be monitored to establish individual baseline seizure frequency as shown in Fig. 3D, and would then each constitute a personalized patient model for use in N-of-1 type testing of compounds to assess antiepileptic efficacy.

Within each animal, seizures were highly stereotyped, both behaviorally (movies S1–4) and electrographically (Fig. 3, fig. S5). Averaged traces of all events demonstrate that each animal’s seizures displayed characteristic morphology and frequency (fig. S5), theoretically facilitating rapid automated analysis of seizure burden in subsequent N-of-1 trials. An interesting pattern also emerged when analyzing seizure event distribution. In the two animals with frequent seizures, events displayed clear circadian rhythmicity (Fig. 3C), with seizures clustering around lights-off (Fig. 3D), the time when mice typically transition to periods of wakefulness (22, 23). This distribution mirrors a characteristic pattern in SeLECTs, wherein seizures most often occur during non-REM sleep or immediately after waking (24), further adding clinical validity as a patient model. Finally, the presence of frequent spontaneous seizures contributes to the model’s utility in assessing patient-specific anti-seizure pharmacotherapy.

## Potential for personalized medicine applications with PegAssist models

Our results provide initial evidence for the feasibility, validity, and utility of in utero genome editing to model an epilepsy patient variant in wildtype mice. The PegAssist approach holds several advantages over other modeling strategies: 1) The use of outbred wildtype animals diminishes cost and time of animal production, and increases genetic and behavioral robustness (25). 2) Since editing in each cell is a distinct event, rare off-target edits are not amplified and are unlikely to influence outcomes, avoiding clonal artifacts that afflict animal-lines (26). 3) The technology used is not species-limiting, meaning a similar approach can be used in non-rodent mammals, including non-human primates.

PE has been successfully applied to a variety of genomic loci in vitro and more recently in vivo (27, 28), suggesting this approach is likely applicable to a wide range of genetic epilepsies. We propose that this pipeline may be a valuable tool for assessing personalized pharmacotherapy options for individual patients, and for preclinical assays for ultra-rare genetic disease in the drug development process.

## Supplementary Material

Supplement 1

Supplement 2

Supplement 3

Supplement 4

Supplement 5

Supplement 6

1

## References

[1] Demarest S. T., Brooks-Kayal A., From molecules to medicines: the dawn of targeted therapies for genetic epilepsies. Nat. Rev. Neurol. 14, 735–745 (2018). 30448857 10.1038/s41582-018-0099-3

[2] Knowles J. K. , Precision medicine for genetic epilepsy on the horizon: Recent advances, present challenges, and suggestions for continued progress. Epilepsia. 63, 2461–2475 (2022). 35716052 10.1111/epi.17332 PMC9561034

[3] Epi25 Collaborative, Chen S., Neale B. M., Berkovic S. F., Shared and distinct ultra-rare genetic risk for diverse epilepsies: A whole-exome sequencing study of 54,423 individuals across multiple genetic ancestries. medRxiv (2023), doi:10.1101/2023.02.22.23286310.

[4] Kwan P., Brodie M. J., Early identification of refractory epilepsy. N. Engl. J. Med. 342, 314–319 (2000). 10660394 10.1056/NEJM200002033420503

[5] Saha K. , The NIH Somatic Cell Genome Editing program. Nature. 592, 195–204 (2021). 33828315 10.1038/s41586-021-03191-1 PMC8026397

[6] Doudna J. A., The promise and challenge of therapeutic genome editing. Nature. 578, 229–236 (2020). 32051598 10.1038/s41586-020-1978-5 PMC8992613

[7] Anzalone A. V., Koblan L. W., Liu D. R., Genome editing with CRISPR-Cas nucleases, base editors, transposases and prime editors. Nat. Biotechnol. 38, 824–844 (2020). 32572269 10.1038/s41587-020-0561-9

[8] Charpentier M. , CtIP fusion to Cas9 enhances transgene integration by homologydependent repair. Nat. Commun. 9, 1133 (2018). 29556040 10.1038/s41467-018-03475-7 PMC5859065

[9] Anzalone A. V. , Search-and-replace genome editing without double-strand breaks or donor DNA. Nature. 576, 149–157 (2019). 31634902 10.1038/s41586-019-1711-4 PMC6907074

[10] Richardson R. R. , Enhancing Precision and Efficiency of Cas9-Mediated Knockin Through Combinatorial Fusions of DNA Repair Proteins. The CRISPR Journal (2023), doi:10.1089/crispr.2023.0036. PMC10611978 37713292

[11] Hsu J. Y. , PrimeDesign software for rapid and simplified design of prime editing guide RNAs. Nat. Commun. 12, 1034 (2021). 33589617 10.1038/s41467-021-21337-7 PMC7884779

[12] Yu G. , Prediction of efficiencies for diverse prime editing systems in multiple cell types. Cell (2023), doi:10.1016/j.cell.2023.03.034. 37119812

[13] Lemke J. R. , Mutations in GRIN2A cause idiopathic focal epilepsy with rolandic spikes. Nat. Genet. 45, 1067–1072 (2013). 23933819 10.1038/ng.2728

[14] García-Recio A. , GRIN database: A unified and manually curated repertoire of GRIN variants. Hum. Mutat. 42, 8–18 (2021). 33252190 10.1002/humu.24141

[15] Salmi M. , Impaired vocal communication, sleep-related discharges, and transient alteration of slow-wave sleep in developing mice lacking the GluN2A subunit of N-methyld-aspartate receptors. Epilepsia. 60, 1424–1437 (2019). 31158310 10.1111/epi.16060

[16] Strehlow V. , GRIN2A-related disorders: genotype and functional consequence predict phenotype. Brain. 142, 80–92 (2019). 30544257 10.1093/brain/awy304 PMC6308310

[17] Amador A. , Modelling and treating GRIN2A developmental and epileptic encephalopathy in mice. Brain. 143, 2039–2057 (2020). 32577763 10.1093/brain/awaa147 PMC7363493

[18] Robasky K., Lewis N. E., Church G. M., The role of replicates for error mitigation in nextgeneration sequencing. Nat. Rev. Genet. 15, 56–62 (2014). 24322726 10.1038/nrg3655 PMC4103745

[19] Paoletti P., Ascher P., Neyton J., High-affinity zinc inhibition of NMDA NR1-NR2A receptors. J. Neurosci. 17, 5711–5725 (1997). 9221770 10.1523/JNEUROSCI.17-15-05711.1997 PMC6573217

[20] Gobbi G., Boni A., Filippini M., The spectrum of idiopathic Rolandic epilepsy syndromes and idiopathic occipital epilepsies: from the benign to the disabling. Epilepsia. 47 Suppl 2, 62–66 (2006). 10.1111/j.1528-1167.2006.00693.x 17105465

[21] Beaussart M., Benign epilepsy of children with Rolandic (centro-temporal) paroxysmal foci. A clinical entity. Study of 221 cases. Epilepsia. 13, 795–811 (1972). 4509173 10.1111/j.1528-1157.1972.tb05164.x

[22] Jin B., Aung T., Geng Y., Wang S., Epilepsy and its interaction with sleep and circadian rhythm. Front. Neurol. 11, 327 (2020). 32457690 10.3389/fneur.2020.00327 PMC7225332

[23] Ripperger J. A., Jud C., Albrecht U., The daily rhythm of mice. FEBS Lett. 585, 1384–1392 (2011). 21354419 10.1016/j.febslet.2011.02.027

[24] Specchio N. , International League Against Epilepsy classification and definition of epilepsy syndromes with onset in childhood: Position paper by the ILAE Task Force on Nosology and Definitions. Epilepsia. 63, 1398–1442 (2022). 35503717 10.1111/epi.17241

[25] Tuttle A. H., Philip V. M., Chesler E. J., Mogil J. S., Comparing phenotypic variation between inbred and outbred mice. Nat. Methods. 15, 994–996 (2018). 30504873 10.1038/s41592-018-0224-7 PMC6518396

[26] Doetschman T., Influence of genetic background on genetically engineered mouse phenotypes. Methods Mol. Biol. 530, 423–433 (2009). 19266333 10.1007/978-1-59745-471-1_23 PMC2805848

[27] Zhi S. , Dual-AAV delivering split prime editor system for in vivo genome editing. Mol. Ther. 30, 283–294 (2022). 34298129 10.1016/j.ymthe.2021.07.011 PMC8753371

[28] Davis J. R. , Efficient prime editing in mouse brain, liver and heart with dual AAVs. Nat. Biotechnol. (2023), doi:10.1038/s41587-023-01758-z. PMC10869272 37142705

[29] Richardson C. D., Ray G. J., DeWitt M. A., Curie G. L., Corn J. E., Enhancing homology-directed genome editing by catalytically active and inactive CRISPR-Cas9 using asymmetric donor DNA. Nat. Biotechnol. 34, 339–344 (2016). 26789497 10.1038/nbt.3481

[30] Saito T., Nakatsuji N., Efficient gene transfer into the embryonic mouse brain using in vivo electroporation. Dev. Biol. 240, 237–246 (2001). 11784059 10.1006/dbio.2001.0439

[31] Poulopoulos A. , Subcellular transcriptomes and proteomes of developing axon projections in the cerebral cortex. Nature. 565, 356–360 (2019). 30626971 10.1038/s41586-018-0847-y PMC6484835

[32] Clement K. , CRISPResso2 provides accurate and rapid genome editing sequence analysis. Nat. Biotechnol. 37, 224–226 (2019). 30809026 10.1038/s41587-019-0032-3 PMC6533916

[33] Borschel W. F. , Gating reaction mechanism of neuronal NMDA receptors. J. Neurophysiol. 108, 3105–3115 (2012). 22993263 10.1152/jn.00551.2012 PMC3544869

[34] Amico-Ruvio S. A., Murthy S. E., Smith T. P., Popescu G. K., Zinc effects on NMDA receptor gating kinetics. Biophys. J. 100, 1910–1918 (2011). 21504727 10.1016/j.bpj.2011.02.042 PMC3077707

[35] Brody D. L., Patil P. G., Mulle J. G., Snutch T. P., Yue D. T., Bursts of action potential waveforms relieve G-protein inhibition of recombinant P/Q-type Ca2+ channels in HEK 293 cells. J. Physiol. (Lond.). 499 (Pt 3), 637–644 (1997). 9130160 10.1113/jphysiol.1997.sp021956 PMC1159282

[36] Hirsch L. J. , American clinical neurophysiology society’s standardized critical care EEG terminology: 2021 version. J Clin Neurophysiol. 38, 1–29 (2021). 33475321 10.1097/WNP.0000000000000806 PMC8135051

