# Supplementary material for: Rapid modeling of an ultra-rare epilepsy variant in wild-type mice by in utero prime editing
Source: bioRxiv. 2023 Dec 19:2023.12.06.570164. Originally published 2023 Dec 8. Preprint. [Version 2] doi: 10.1101/2023.12.06.570164 (PMC10723435; doi:10.1101/2023.12.06.570164)
Supplement: Supplement 7 [file NIHPP2023.12.06.570164v2-supplement-7.pdf]

bioRxiv preprint doi: https://doi.org/10.1101/2023.12.06.570164; this version posted December 19, 2023. The copyright holder for this preprint (which was not certified by peer review) is the author/funder, who has granted bioRxiv a license to display the preprint in perpetuity. It is made available under aCC-BY 4.0 International license.

Supplementary Table S1. Individual annotation of seizures and epileptiform events ("Events" tab), and details of PA and CT animal cohorts ("Cohorts" tab).

Supplementary Table S2. Plasmids and oligonucleotides used: identifiers, purpose, and sequences.

Supplementary Videos S1-2. Videos of seizures corresponding to the two example traces from animal PA2 in Fig. 4a.

Supplementary Videos S3-4. Videos of seizures corresponding to the two example traces from animal PA5 in Fig. 4a.
