# Supplementary material for: Modeling an ultra-rare epilepsy variant in wildtype mice with in utero prime editing
Source: bioRxiv. 2023 Dec 8:2023.12.06.570164. Preprint. [Version 1] doi: 10.1101/2023.12.06.570164 (PMC10723435; doi:10.1101/2023.12.06.570164)
Supplement: Supplementary file 1 [file NIHPP2023.12.06.570164V1-supplement-1.pdf]

bioRxiv preprint doi: https://doi.org/10.1101/2023.12.06.570164; this version posted December 8, 2023. The copyright holder for this preprint (which was not certified by peer review) is the author/funder, who has granted bioRxiv a license to display the preprint in perpetuity. It is made available under aCC-BY 4.0 International license.

Supplementary Materials for

Modeling an ultra-rare epilepsy variant in wildtype mice with in utero prime editing

Colin D. Robertson[1], Patrick Davis[2], Ryan R. Richardson[1], Philip H. Iffland II[3], Daiana C. O. Vieira[4], Marilyn Steyert[1]†, Paige N. McKeon[1], Andrea J. Romanowski[1], Garrett Crutcher[1], Eldin Jašarević[1]†, Steffen B. E. Wolff[1], Brian N. Mathur[1], Peter B. Crino[3], Tracy L. Bale[1]†, Ivy E. Dick[4], Alexandros Poulopoulos[1]*

[1]Department of Pharmacology and UM-MIND, University of Maryland School of Medicine, Baltimore, MD, USA

[2]Department of Neurology, Boston Children's Hospital, Harvard Medical School, Boston, MA, USA

[3]Department of Neurology, and UM-MIND, University of Maryland School of Medicine, Baltimore, MD, USA

[4]Department of Physiology and UM-MIND, University of Maryland School of Medicine, Baltimore, MD, USA

*Corresponding author. Email: apoulopoulos@som.umaryland.edu

†Current affiliations: MS: Department of Neurological Surgery, University of California San Francisco; EJ: Department Computational and Systems Biology, Department of Obstetrics, Gynecology and Reproductive Sciences, University of Pittsburgh School of Medicine; TB: Department of Psychiatry, University of Colorado School of Medicine.

Materials included:

Materials and Methods
Figs. S1 to S5
Table S1 to S2 descriptions
Movie S1-S4 descriptions

bioRxiv preprint doi: https://doi.org/10.1101/2023.12.06.570164; this version posted December 8, 2023. The copyright holder for this preprint (which was not certified by peer review) is the author/funder, who has granted bioRxiv a license to display the preprint in perpetuity. It is made available under aCC-BY 4.0 International license.

Materials and Methods

pegRNA design and PegAssist application

To facilitate the use and broad adoption of prime editing, we developed PegAssist and an accompanying webtool and set of plasmids for the design and production of custom Prime editing reagents. The python-based web application pegassist.app accepts input of target sequence and desired edits to produce pegRNA sequences and one-step cloning strategies based on the PegAssist plasmid set. The PegAssist platform offers PE2, PE3 and PE3b variant strategies, and further allows custom modifications of spacers and PAM sequences for versatility and use with further developments in genome editors (fig. S2).

The PegAssist python source code is available on github.com/pegassist. A graphical user interface was created using Heroku to compile a webtool available at pegassist.app.

To generate *Grin2a*-A243V pegRNAs, a custom spacer (20 nt) was first designed to minimize the distance between the protospacer adjacent motif (PAM) site and the edit position. The custom spacer and desired edit were used to generate automated reverse transcriptase template and primer binding sequence options and secondary nicking (PE3/PE3b) guides following the instructions outlined by the webtool. Preference was given to PBS ~13nt with >30% GC-content and RT <20nt. PE3b guides were selected to minimize unintended indels.

Plasmids

Double-stranded DNA oligonucleotides containing pegRNAs flanked by BbsI recognition sites were synthesized by Twist Bioscience. Sense and antisense oligonucleotides for knockout gRNA or PE3b gRNA with overhangs for golden gate assembly were synthesized by Integrated Design Technologies. Cloning was performed as previously described (*10*). Briefly, pegRNA oligonucleotides were sub-cloned into pCR Blunt II-TOPO backbone (ThermoFisher). A golden gate assembly (GGA) with BbsI was used to clone the pegRNA into a custom backbone containing a hU6 promotor. The PE3/PE3b or knockout gRNA sense and antisense oligonucleotides were annealed and cloned by GGA into a pJ2 (*10*) containing a hU6 promoter. The vectors containing pegRNA and PE3/PE3b guides were used as template for PCR using KAPA HiFi HotStart DNA Polymerase with 2x Master Mix (Roche) to amplify parts containing the U6 promoter and either the pegRNA or PE3/PE3b guide using primers with BsaI recognition sites. These parts were assembled in a final vector by golden gate assembly using NEB Golden Gate Assembly Kit according to manufacturer's recommendations. The open reading frame of PE2 was extracted from pCMV-PE2 (Addgene plasmid #132775) by PCR with primers (prRR842 and prRR849). PE2 was introduced into a pJ2 backbone by GGA to construct the final plasmid pJ2.CAG<EGFP-2A-PE2 [Lab plasmid ID: TU516]. Plasmid pCAG<myr-tdTomato expressing myristoylated tdTomato was subcloned from Addgene plasmid #26771 and used as a bright fluorescence electroporation reporter. Plasmid sequences were confirmed by Sanger sequencing by GeneWiz. Details and sequences of plasmids and oligonucleotides used are listed in table S2.

BFP-to-GFP conversion assay

bioRxiv preprint doi: https://doi.org/10.1101/2023.12.06.570164; this version posted December 8, 2023. The copyright holder for this preprint (which was not certified by peer review) is the author/funder, who has granted bioRxiv a license to display the preprint in perpetuity. It is made available under aCC-BY 4.0 International license.

BFP-to-GFP conversion assays were performed as previously described (*10*). Briefly, a modified HEK-293 cell line with genomically-encoded BFP was a gift from the Corn lab (*29*). Cells were maintained in DMEM with GlutaMAX (ThermoFisher Scientific) supplemented with 10% fetal bovine serum. Cells were plated at a density of 20,000-22,500 cells/cm^2 in 24-well plates prior to transfection using polyethylenimine, linear, MW 25000 (Polysciences) at 1 mg/mL in diH_2O, then mixed in a 3:1 ratio with 750 ng total DNA diluted in Opti-MEM per well.

Conversion of BFP to GFP was analyzed by flow cytometry using an LSRII cell analyzer with HRS (BD Biosciences). A 407 nm laser with a 405/50 emission filter was used to detect BFP, while a 488 nm laser with a 505 LP mirror and a 530/30 emission filter was used to measure GFP.

Mice

Experimental protocols involving animals were approved by the University of Maryland Baltimore Institutional Animal Care and Use Committee. Pregnant, outbred CD1 mice were obtained from Charles River Laboratories. *In utero* electroporation was performed on embryonic mice ambiguous to considerations of sex. Mice were weaned at P21 and EEG/EMG recordings were performed at 3-8 months.

In utero electroporation

Cortical layer II/III pyramidal neurons were targeted by performing this procedure *in utero* on embryonic day 14.5 as previously described (*30*, *31*). Briefly, plasmid DNA was combined to a maximum concentration of 4 μg/μL with equal molar ratios of relevant plasmids (pegRNA/PE3b duplex, prime editor, and fluorescent reporter). Dams were anesthetized with isoflurane with thermal support. The abdomen was prepared for surgery by removing hair and sanitizing the incision site using betadine and 70% ethanol. An incision of the skin and muscle layer along the midline exposed the uterine horns. A glass micropipette was pulled (Narishige PC-100) and beveled (Narishige PCR-45). The micropipette was attached to an aspirator and used to inject the prepared DNA mixture into the right ventricle of developing fetuses. Immediately upon injection, a series of 4 x 50 ms square pulses of 35 V (NEPA21 electro-kinetic platinum tweezertrodes on a BTX ECM-830 electroporator) was used to introduce the DNA into neural progenitor cells lining the ventricle. In a typical surgery 3-6 pups were electroporated. Following electroporation, the uterine horn was returned to the abdominal cavity, and the muscle and skin layers were closed using monofilament nylon sutures (AngioTech). After birth, electroporated pups were screened at post-natal day 0 for fluorescence using a fluorescence stereoscope (Leica MZ10f with X-Cite Fire LED light source). Positively screened pups were returned to the dam.

Neuron Fluorescence Activated Cell Sorting and Next-Generation Sequencing

In utero electroporated mice were deeply anesthetized using isofluorane and euthanized. The brain was removed and immediately moved to pre-cooled dissociation medium (20 mM glucose, 0.8 mM kynurenic acid, 0.05 mM APV, 50 U/ml penicillin, 0.05 mg/mL streptomycin, 0.09 M Na_2SO_4, 0.03 M K_2SO_4, 0.014 M $MgCl_2$) on ice. Using a fluorescence stereoscope (Leica MZ10f with X-Cite Fire LED light source), the electroporated region was dissected and transferred to a new tube containing ice-cold dissociation medium. Dissociation medium was

bioRxiv preprint doi: https://doi.org/10.1101/2023.12.06.570164; this version posted December 8, 2023. The copyright holder for this preprint (which was not certified by peer review) is the author/funder, who has granted bioRxiv a license to display the preprint in perpetuity. It is made available under aCC-BY 4.0 International license.

aspirated until 1 mL remained and an activated papain solution (1:1 papain [Worthington-Biochem] with 13.6 mM Cysteine-HCL, 0.002% β-mercaptoethanol, and 2.4 mM EDTA pH 8.0 in MilliQ water) was added and incubated at 37° C for 30 minutes. Papain solution was removed, and tissue was washed three times and resuspended in 500 µL fresh dissociation medium. Samples were triturated 2-4 times using flame-polished borosilicate pipettes. Cell suspensions were sorted at low flow rates using a Wolf Benchtop Cell Sorter (Nanocollect Biomedical, Inc.) using red fluorescence from pCAG<myr-tdTomato for gating, after confirming overlap with green fluorescent signal from co-expressed pJ2.CAG<EGFP-2A-PE2. Approximately 3,000 cells were collected per sample. Sorted cells were pelleted and stored at -80°C.

Cells were lysed and total RNA was extracted using an AllPrep DNA/RNA Micro Kit (Qiagen). A reverse transcription reaction using a First Strand cDNA Synthesis Kit (Millipore Sigma) was used to create cDNA from the isolated RNA. The region surrounding the intended edit was amplified by PCR using primers containing universal adaptor sequences (prCR419 and prCR420). These amplicons were submitted to the Institute for Genome Sciences at the University of Maryland School of Medicine for sequencing, where samples were quantified, barcoded and sequenced on an Illumina NextSeq 550 (Illumina) according to manufacturer settings. An average of 8.9 million reads per sample were analyzed. GRCm39 was used as reference genome. Sanger sequencing of the target region from CD1 mice used in experiments were consistent with the reference genome (data not shown). Sequencing results were analyzed using CRISPResso2 (*32*) under prime editing mode. Default CRISPResso2 parameters were applied (quantification window 10, nicking guide sequence defined, scaffold match length 1). A contiguous quantification window was produced encompassing both pegRNA and PE3b gRNA target sequences.

Electrophysiology

Whole-cell currents were recorded at room temperature from mouse cortical neurons which were isolated at P0 from E15 in utero electroporated animals and cultured 30 days in vitro (DIV) in order to allow for expression of GluN2A, which is known to be developmentally regulated (*33*). Only cells expressing the red fluorescent marker were patched. Electrodes were pulled from borosilicate glass capillaries that were fire-polished to a resistance of 3-4 MΩ and filled with intracellular solution (mM): 135 CsCl, 35 CsOH, 4 MgATP, 0.3 Na_2GTP, 10 HEPES and 1 EGTA, adjusted to pH 7.4 with CsOH. Cells were perfused with extracellular solutions containing (mM): 140 NaCl, 2.5 KCl, 1.8 $CaCl_2$, 0.1 glycine, 10 HEPBS, 10 tricine, adjusted to pH 7.4 (NaOH). 40 µM cyanquixaline (CNQX) and 1µM ifenprodil were added to external solution to block GluN2B and AMPA receptors respectively. For solutions containing zinc, free Zn^{2+} concentrations in 10 mM tricine-buffered solutions were calculated using Maxchelator software (Chris Patton) using a binding constant of 10^{-5} M as previously reported (*19*) and adjusted for our conditions. The final free zinc concentration was chelated to 67 nM by adding 200 mM $ZnCl_2$ and 10 mM tricine into the working extracellular solution (*34*). Currents were recorded with an Axopatch 200B amplifier (Molecular Devices) and digitized using an iTC-18 (InstruTECH). Currents were low-pass filtered at 2 kHz and sampled at 10 kHz using custom MATLAB (MathWorks) scripts. Drugs and agonists were applied during the patch recordings by means of an eight-barrel pen-perfusion system, with minimal dead space. In all whole-cell

bioRxiv preprint doi: https://doi.org/10.1101/2023.12.06.570164; this version posted December 8, 2023. The copyright holder for this preprint (which was not certified by peer review) is the author/funder, who has granted bioRxiv a license to display the preprint in perpetuity. It is made available under aCC-BY 4.0 International license.

experiments, the cells were clamped at -80 mV. Solution containing NMDA (100 μM), or NMDA with 67 nM free Zn^{2+} was applied to elicit the current, usually for 5 sec every 2 min, using motor-driven valves.

For HEK 293 cell recordings, cells were cultured on glass coverslips coated with poly-D-lysine and transfected via the calcium phosphate method (*35*) with 4–8 μg of rat GluN1-1a and GluN2A or GluN2A (A243V), and co-transfected with 2 μg green fluorescent protein. Culture media was exchanged 3–5 h post-transfection, and cells were maintained 24–48 hours in DMEM supplemented with 2 mM Mg^{2+} to prevent NMDA receptor-mediated cell death. The GluN1-1a and GluN2A plasmids were a kind gift from Gabriela Popescu (University at Buffalo). The mutation A243V was introduced into the GluN2A plasmid using the QuickChange II XL kit from Agilent. All portions of the resulting construct that were subject to PCR were confirmed by DNA sequencing. Recording conditions were identical to those listed above for neurons.

<u>Continuous EEG/EMG recording</u>

Synchronous EEG/EMG and video recording was performed using a tethered, 3-channel recording system from Pinnacle Technology Inc. Prefabricated EEG headmounts were implanted with screw electrodes 3 mm behind Bregma and an EMG lead was implanted in the trapezius muscle of mice under isofluorane anesthesia. Mounted electrodes were fixed using dental epoxy. After minimum 72 hours recovery, mice were placed in recording chambers for synchronous EEG/EMG and video recording for approximately 4 days.

The EEG/EMG data were exported and blinded before primary review. An initial investigator manually annotated possible epileptiform events and exported epochs for secondary review by a blinded expert (author PFD) and classification based on the American Clinical Neurophysiology Society's most recent standardized criteria (*36*). Specifically, seizure was defined as any rhythmic epileptiform discharge with either a) time-locked, consistent behavioral correlate, b) discharges averaging >2.5Hz for at least 10s, or c) with definite spatiotemporal evolution and lasting at least 10s. Based on accepted ACNS criteria for human EEG, the latter two categories would be termed "electrographic seizure" as opposed to "electroclinical seizure", but in this analysis we did not make such a distinction.

EEG, EMG, and video data were used to visually identify artifacts associated with movement. If movement associated with EEG change was stereotyped across events (within animals) (see videos S1-4), the abnormal movements associated with these events were considered clinical correlates. Seizure events were further classified as lateralized for events occurring in only one EEG channel or generalized for events with synchronous activity between both EEG channels. If similar activity was seen that was without clear clinical correlate, did not last 10s duration, and did not have clear spatiotemporal evolution, these events were classified as interictal discharges ("epileptiform" in table S1 and fig. S5) as opposed to seizures. Seizure epoch charts were generated from annotated seizure bouts using MNE-Python following standardization using the SciKit StandardScaler function. Seizure frequency was calculated as the total number of events divided by days of recording.

<u>Tissue Fixation and Immunolabeling</u>

bioRxiv preprint doi: https://doi.org/10.1101/2023.12.06.570164; this version posted December 8, 2023. The copyright holder for this preprint (which was not certified by peer review) is the author/funder, who has granted bioRxiv a license to display the preprint in perpetuity. It is made available under aCC-BY 4.0 International license.

Tissue was fixed by transcardial perfusion of mice using PBS and 4% paraformaldehyde with 24 hours post-fixation in 4% paraformaldehyde at 4°C. Tissue slices were cut to 80 μm using a vibrating microtome (Leica). For immunolabeling, slices were incubated in a blocking solution of 5% BSA, 0.3% TritonX-100, and 0.05% sodium azide in PBS for 2 hours while rocking at room temperature. Primary antibodies were diluted 1:1000 in blocking solution, and tissue was incubated overnight, rocking at room temperature. Slices were washed 3x 30 minutes in PBS while rocking, then incubated with secondary antibody at 1:1000 in PBS at room temperature for 4 hours. After 3x 30-minute PBS washes the slices were mounted on slides with either Fluoromount-G Mounting Medium with or without DAPI (ThermoFisher Scientific).

Microscopy

Fluorescence images were acquired using a Nikon Ti2-E inverted epifluorescence microscope. Images were analyzed using NIS Elements (Nikon). Proximal z-stacks were acquired using a 10x objective, then extended depth of focus and stitching were used to compile a single slice image.

bioRxiv preprint doi: https://doi.org/10.1101/2023.12.06.570164; this version posted December 8, 2023. The copyright holder for this preprint (which was not certified by peer review) is the author/funder, who has granted bioRxiv a license to display the preprint in perpetuity. It is made available under aCC-BY 4.0 International license.

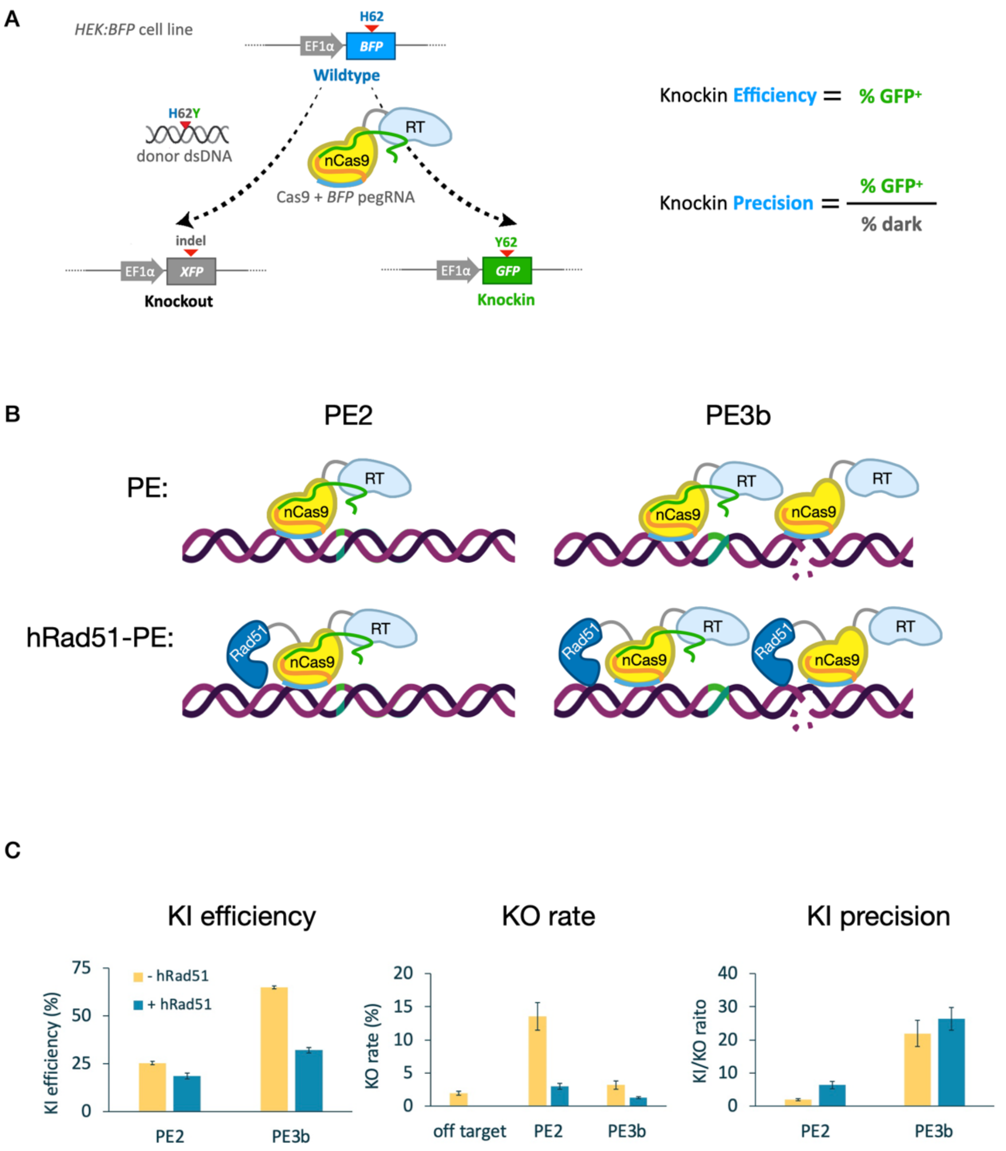

Fig. S1.

Screening editing agents for precision identifies prime editing with PE3b as highest precision for point editing. (**A**) Schematic (adapted[10]) depicting the BFP-to-GFP conversion assay. HEK cells with genomic knockin of BFP were transfected with editing agents to introduce H62Y edit, which corresponds to the sequence of GFP. Precise editing converts cells from blue to green, while imprecise edits cause loss of fluorescence. Editing precision is calculated as the ratio of green/dark cells. (**B**) Schematic of the editing approaches tested with the assay. Standard PE2 and PE3b approaches were tested in combination with an hRad51-fused prime editor. (**C**) Quantification of knockin efficiency, knockout rate, and knockin precision. Addition of hRad51 improved PE2 strategy's precision but did not significantly improve PE3b. The standard PE3b approach provided the highest efficiency and precision.

bioRxiv preprint doi: https://doi.org/10.1101/2023.12.06.570164; this version posted December 8, 2023. The copyright holder for this preprint (which was not certified by peer review) is the author/funder, who has granted bioRxiv a license to display the preprint in perpetuity. It is made available under aCC-BY 4.0 International license.

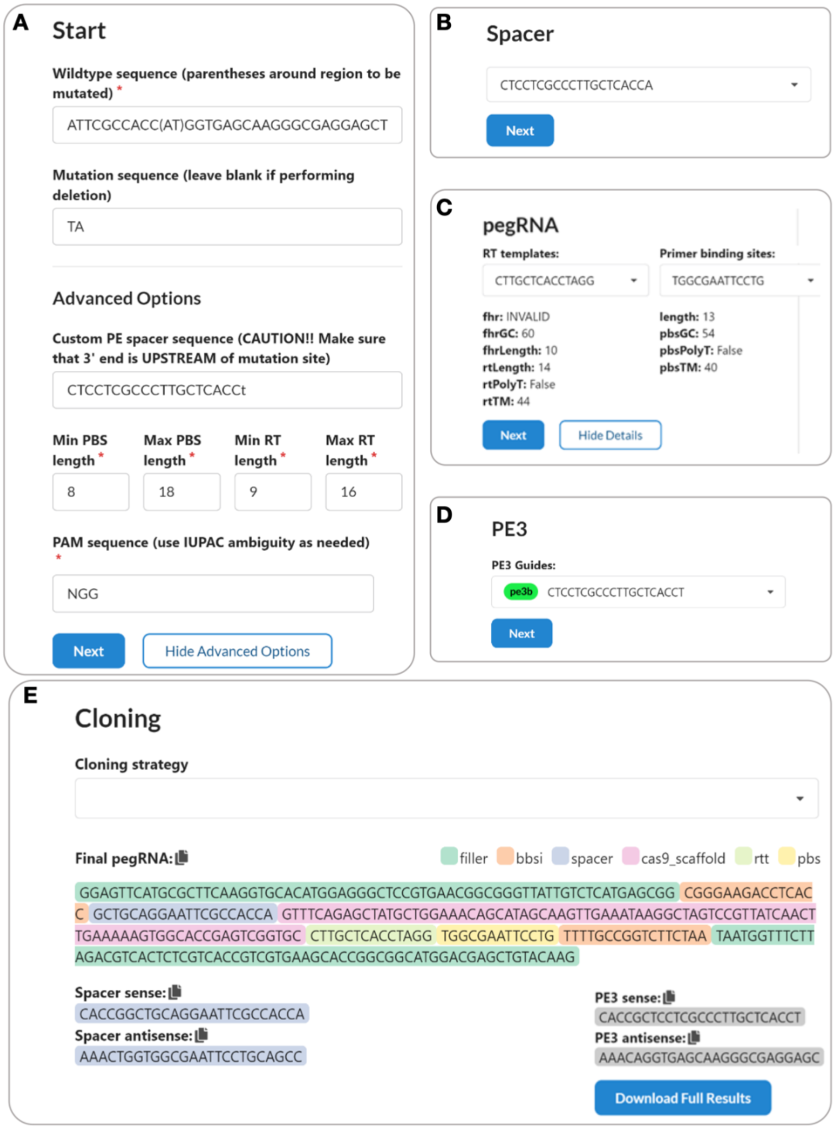

Fig. S2.

pegassist.app simplifies pegRNA and PE3b guide design. (**A**) pegassist.app accepts input of a wildtype sequence with the desired edit in parentheses. The edited sequence is entered, and users can select from additional options including input of a pre-designed spacer sequence. (**B**) A spacer sequence is selected from the dropdown menu. (**C**) RT template and Primer binding site options are presented with warnings against low efficiency. (**D**) All PE3/PE3b options are presented for selection. (**E**) pegassist.app displays a final pegRNA and the required oligos based on the selected cloning method.

bioRxiv preprint doi: https://doi.org/10.1101/2023.12.06.570164; this version posted December 8, 2023. The copyright holder for this preprint (which was not certified by peer review) is the author/funder, who has granted bioRxiv a license to display the preprint in perpetuity. It is made available under aCC-BY 4.0 International license.

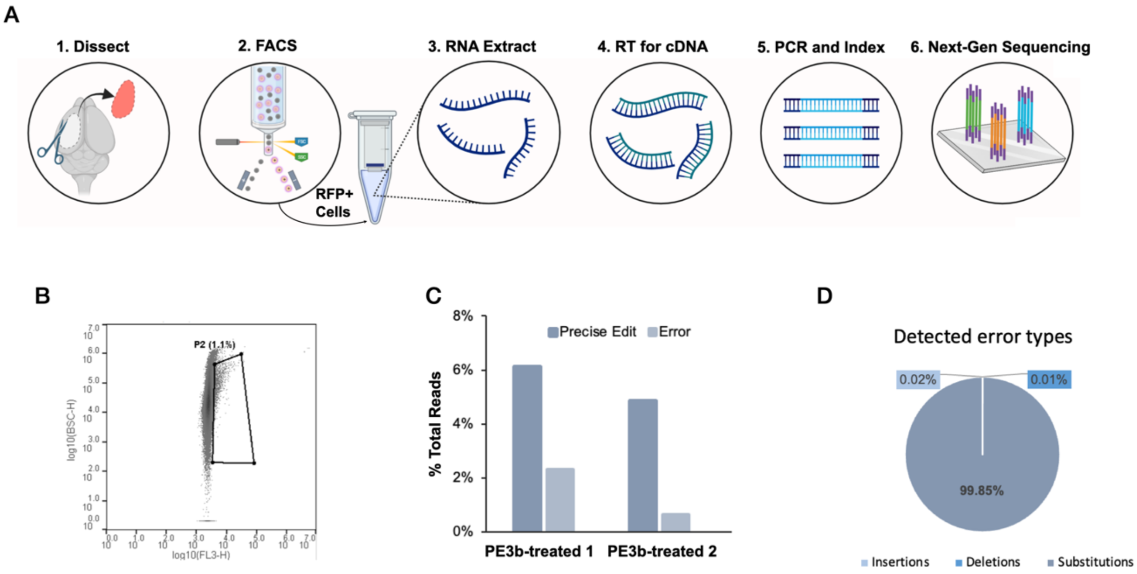

Fig. S3.

In vivo prime editing of *Grin2a* is precise and causes few indels. (**A**) Schematic representation of the sequencing workflow. Following in utero electroporation with PE3b agents, the PE-expressing region of cortex was dissected from adult mice. Cells were triturated and sorted for red fluorescence. RNA was extracted to generate cDNA by reverse transcription. Target sequence was amplified by PCR. Amplicons were indexed and sequenced on the Illumina NextSeq platform. (**B**) FACS plot of neurons gated to collect electroporated red fluorescent neurons (~1% of total neurons). y axis plots side scatter and x axis plots red fluorescence intensity. (**C**) Percentage of reads with either the intended edit or any error in the two PE3b-treated animals sequenced. The intended edit is detected approximately 5 times more frequently than the aggregate of all proximal erroneous edits. (**D**) Breakdown of error types detected in PE3b-treated samples. Most errors are substitutions and may be expected to have less deleterious effects on the resulting protein than indels.

bioRxiv preprint doi: https://doi.org/10.1101/2023.12.06.570164; this version posted December 8, 2023. The copyright holder for this preprint (which was not certified by peer review) is the author/funder, who has granted bioRxiv a license to display the preprint in perpetuity. It is made available under aCC-BY 4.0 International license.

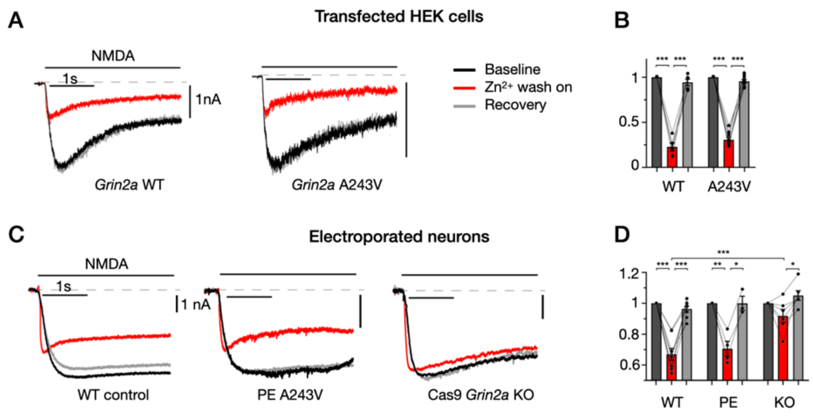

Fig. S4.
Electrophysiology shows *Grin2a* KO is not a common outcome of in utero PE3b. (**A**) Example traces (black=baseline, red=zinc wash-on, grey=recovery) and (**B**) normalized (within sample to baseline) amplitudes of currents recorded from HEK cells co-transfected with *GRIN1* and either WT (n=5) or A243V (n=8) variant of *GRIN2A*. No change in the sensitivity to Zn2+ blockade of NMDA currents. (**C**) Example traces and (**D**) normalized (within sample to baseline) amplitudes of currents recorded from in utero electroporated neurons after cultured after in utero electroporation. Zinc blockade was detected in Sham (n=8) and PE *Grin2a-A243V* cells (n=4), but not KO cells (n=6), indicating that treatment with PE3b does not cause significant loss-of-function (*p<0.05, ***p<0.001, via students T-test).

bioRxiv preprint doi: https://doi.org/10.1101/2023.12.06.570164; this version posted December 8, 2023. The copyright holder for this preprint (which was not certified by peer review) is the author/funder, who has granted bioRxiv a license to display the preprint in perpetuity. It is made available under aCC-BY 4.0 International license.

Fig. S5

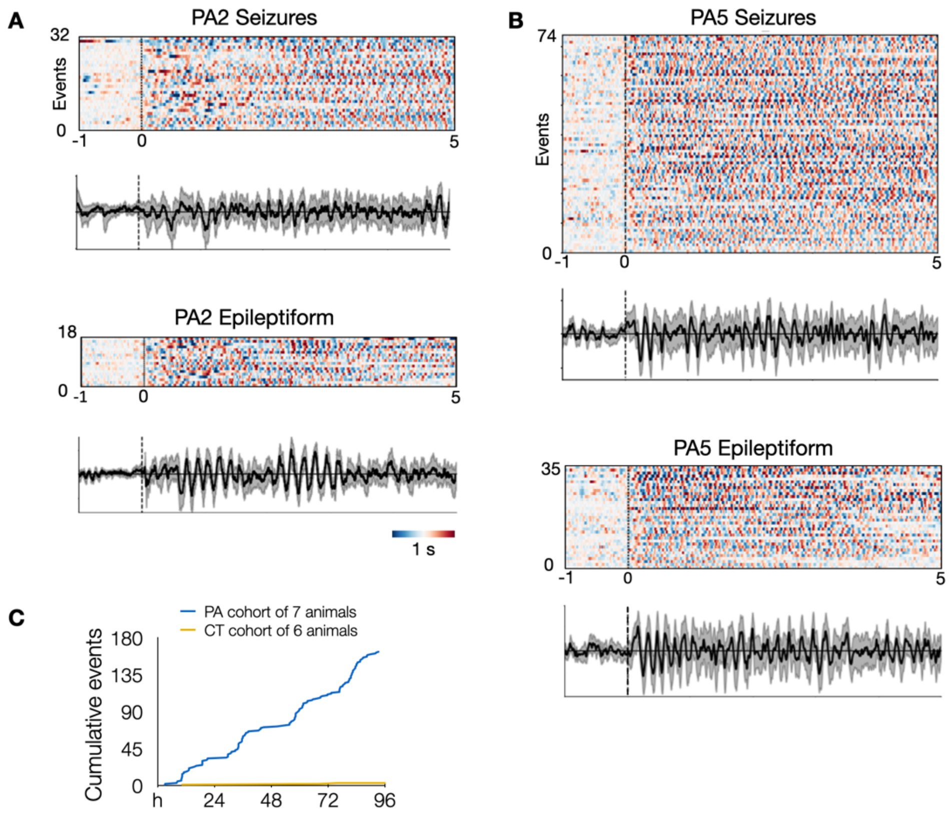

Fig. S5.

Epoch charts and averaged traces show the stereotyped nature of seizures and epileptiform events. (**A**) PA2 and (**B**) PA5 epoch charts and averaged traces for events classified as seizures or epileptiform events. Each row represents a separate event aligned at onset of abnormal activity (t = 0) and plotted from t = -1 s to t = 5 s. Color bar indicates standardized EEG amplitude from peak negative (dark blue) to peak positive (dark red). Traces show an averaged EEG signal in black with bootstrapped 95% confidence intervals in grey. Charts were generated using MNE-Python. (**C**) Pooled cumulative events (including both seizures and epileptiform events) between PA and CT groups over 4 days of EEG recording. Heatmap and scale bar = 1 s.

bioRxiv preprint doi: https://doi.org/10.1101/2023.12.06.570164; this version posted December 8, 2023. The copyright holder for this preprint (which was not certified by peer review) is the author/funder, who has granted bioRxiv a license to display the preprint in perpetuity. It is made available under aCC-BY 4.0 International license.

Table S1.

Individual annotation of seizures and epileptiform events ("Events" tab), and details of PA and CT animal cohorts ("Cohorts" tab).

Table S2.

Plasmids and oligonucleotides used: identifiers, purpose, and sequences.

Movie S1.

Videos of seizures corresponding to the first example trace from animal PA2 in Fig. 2A.

Movie S2.

Videos of seizures corresponding to the second example trace from animal PA2 in Fig. 2A.

Movie S3.

Videos of seizures corresponding to the first example trace from animal PA5 in Fig. 2A.

Movie S4.

Videos of seizures corresponding to the second example trace from animal PA5 in Fig. 2A.
